# In silico and in vivo splicing analysis of *MLH1 *and *MSH2 *missense mutations shows exon- and tissue-specific effects

**DOI:** 10.1186/1471-2164-7-243

**Published:** 2006-09-22

**Authors:** Patrizia Lastella, Nicoletta Concetta Surdo, Nicoletta Resta, Ginevra Guanti, Alessandro Stella

**Affiliations:** 1 Section of Medical Genetics, Department of Biomedicine in Childhood, University of Bari, Italy. Policlinico P.zza G.Cesare 11 70124 Bari, Italy

## Abstract

**Background:**

Abnormalities of pre-mRNA splicing are increasingly recognized as an important mechanism through which gene mutations cause disease. However, apart from the mutations in the donor and acceptor sites, the effects on splicing of other sequence variations are difficult to predict. Loosely defined exonic and intronic sequences have been shown to affect splicing efficiency by means of silencing and enhancement mechanisms. Thus, nucleotide substitutions in these sequences can induce aberrant splicing. Web-based resources have recently been developed to facilitate the identification of nucleotide changes that could alter splicing. However, computer predictions do not always correlate with in vivo splicing defects. The issue of unclassified variants in cancer predisposing genes is very important both for the correct ascertainment of cancer risk and for the understanding of the basic mechanisms of cancer gene function and regulation. Therefore we aimed to verify how predictions that can be drawn from in silico analysis correlate with results obtained in an in vivo splicing assay.

**Results:**

We analysed 99 hMLH1 and hMSH2 missense mutations with six different algorithms. Transfection of three different cell lines with 20 missense mutations, showed that a minority of them lead to defective splicing. Moreover, we observed that some exons and some mutations show cell-specific differences in the frequency of exon inclusion.

**Conclusion:**

Our results suggest that the available algorithms, while potentially helpful in identifying splicing modulators especially when they are located in weakly defined exons, do not always correspond to an obvious modification of the splicing pattern. Thus caution must be used in assessing the pathogenicity of a missense or silent mutation with prediction programs. The variations observed in the splicing proficiency in three different cell lines suggest that nucleotide changes may dictate alternative splice site selection in a tissue-specific manner contributing to the widely observed phenotypic variability in inherited cancers.

## Background

The precision and correctness of intron removal during pre-mRNA splicing rely on the recognition of several discrete elements some of which, as the splicing donor and acceptor sites, are mostly invariant. However, many other loosely defined cis-acting elements such as the polypyrimidine tract, the branch site and several other sequences, both exonic and intronic, may contribute to exon recognition. Recently, several reports have shown that exonic sequences are able to regulate splicing proficiency, and that nucleotide substitutions in these sequences, lead or may lead to abnormal splicing or exon skipping [1,2]. Moreover, it has been demonstrated that aberrant splicing can occur as a consequence of mutations that disrupt exonic splicing enhancers (ESEs) or create exonic splicing suppressors (ESSs) [reviewed in [3]]. Exonic splicing enhancers have been identified on the basis of exon mutations that block splicing, of computational comparison of exon sequences, and of the selection of sequences that activate splicing or that bind to specific regulatory proteins, most notably the SR (serine-arginine rich) proteins. Three web-based resources, ESEfinder [4,5], Rescue-ESE [6,7], and PESX [8,9] have recently been developed to identify putative ESEs responsive to the human SR proteins and to predict whether exonic mutations disrupt such elements. These algorithms have identified ESEs that tend to colocalize with natural enhancers, and more frequently in exonic sequences rather than in introns. In a recent review, more than 50 nucleotide substitutions, that had previously been reported to cause exon skipping in vivo, were found to reduce or abolish at least one of these computer-identified ESEs [10]. Therefore, a significant number of disease-associated point mutations or polymorphisms may lead to aberrant splicing. However, enhancer and silencer elements can be juxtaposed in specific exonic regions. Thus, efficient splicing is the result of a plethora of quite complex interactions mediated by different splicing factors, each binding to its proper target sequence. We have recently investigated several mutations altering splicing in the *MLH1 *gene whose mutations are responsible for Hereditary Non Polyposis Colorectal Cancer (HNPCC, MIM 114500) [11,12]. Patients with HNPCC usually have a family history of early onset of synchronous and metachronous colorectal cancers and an elevated risk of several other extracolonic malignancies, mainly of the endometrium, stomach, hepato-biliary tract and ovary. The disease is caused by germline mutations of genes within the DNA mismatch repair (MMR) pathway. Nearly 90% of families with an identified genetic defect harbor mutations in either *MLH1 *(MIM 120436) or *MSH2 *(MIM 609309), [13,14]. The InSIGHT database [15], accessed in March 2003, lists a total of 382 different *MLH1 *and *MSH2 *mutations. While the majority of these are either nonsense or frameshifting alterations with an obvious pathogenic influence on the resultant protein, 26% of the alterations listed in the database are missense mutations and therefore their consequences on biological functions are assumed to rely on the principle that the single change introduced in the amino acid sequence impairs the biological function or the structure of the encoded protein. A recent work [16] has demonstrated that pathogenic missense mutations in the hMLH1 and hMSH2 genes, in contrast to polymorphic variants, tend to colocalize in ESE sequences.

On the basis of all the above evidence, we decided to evaluate the 99 hMLH1/hMSH2 missense mutations listed in the InSIGHT database with the currently available ESE prediction programs. We next investigated the consequences of 20 exonic missense mutations with different predicted effects on putative enhancer and suppressor sequences. We found that the splicing behaviour of these mutations cannot be evaluated only on the basis of their predicted localization in ESE sequences.

To further extend our analysis, we assessed the effects of the 20 missense mutations in three different mammalian cell lines. We observed that some nucleotide changes affect splicing with a different degree of severity in different cellular backgrounds.

## Results

### ESEfinder, RescueESE and PESX identify non overlapping ESE motifs

**Figure 1:**
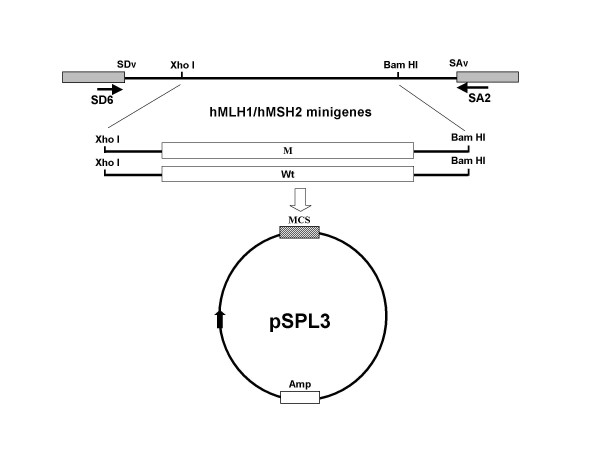
**Schematic representation of the reporter construct used in the splicing assay**. Structure of the chimeric minigene used in all the transfection experiments. Patterned and white boxes indicate the pSPL3 HIV-tat exonic sequences and the human hMLH1/hMSH2 sequences. Arrows show the primers used in the RT-PCR experiments. The indicated XhoI and BamHI sites are those used to clone all the constructs. MCS = multi cloning site, Amp = ampicillin resistance gene, M = mutated construct, Wt = corresponding normal exon.

A recent survey of all the missense mutations and neutral polymorphisms reported in the InSIGHT mutation database for the hMLH1 and hMSH2 genes [15] indicates that missense mutations, but not neutral polymorphisms, tend to occur where ESE sequences are localized [16]. Of the 99 different missense mutations reported in this paper, 50 were localized in ESE sequences identified by ESE finder. We analyzed the same mutation data set with RescueESE and PESX that found respectively 40 and 41 mutations as lying in ESE sites [see additional file 1]. A total of 7 mutations were identified as lying in ESE sites by all the three algorithms. Among these, only 2 caused the same type of predicted change (ie all programs predicted no change, or creation/addition of novel ESE motifs, or disruption of ESE sites). However, in only one case all the three algorithms equally predicted ESE sites disruption without the concurrent creation of novel ESE sites. Since in our previous work we found that the abrogation of ESE motifs identified by the first two algorithms does not always lead to a splicing defect [12], we selected 20 hMLH1 and hMSH2 mutations with different predicted effects on ESEs (table 1). We examined their consequences in a splicing assay we already had available [11] and previously reported to faithfully recapitulate in vivo splicing [17-19]. The criteria for selection were that the mutations should create or abolish one or more ESE sites according to the predictions of at least one algorithm. All the minigene constructs were assembled in the pSPL3 vector (for details see fig. 1 and methods).

### Analysis of 20 hMLH1 and hMSH2 missense mutations shows that mutations altering splicing are situated preferentially in exon subjected to alternative splicing

**Figure 2:**
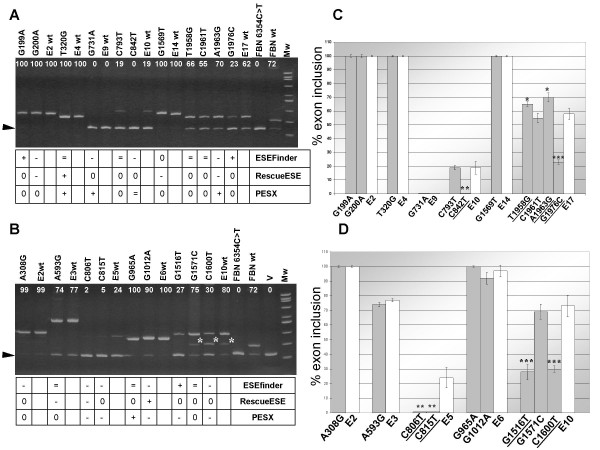
**Effects of the hMLH 1 and hMSH2 mutations on exon inclusion**. Results of the splicing assay with the different hMLH1(A) and hMSH2(B) mutated constructs. Numbering is relative to the nucleotide position in the ORF. Cos-7 cells were transfected with 1 μg of the indicated mutant minigene variants or the corresponding wild-type exon, RNA was extracted, reverse transcribed, and amplified with primers SD6 and SA2. The RT-PCR products were resolved on GeneGel Excel, stained with ethidium bromide and quantitated with an image analyzer (see methods). V = vector only; Mw = size standard. The black arrowhead represents the exon skipped product. The percentage of exon inclusion is indicated above each lane. The white asterisks show the splicing product deriving from use of an internal cryptic donor site. Below the gel are reported the predictions for the three algorithms: = no change; + the mutation creates an ESE or abrogates an ESS sequence; - the mutation creates an ESS or abrogates an ESE sequence; 0 the mutations is not localised in, and does not create or disrupt any regulatory sequence. (C, D) Graphic representation of the splicing assay results. The average of percent exon inclusion is reported in the y-axis and represents the mean of two independent transfections done in triplicate for each construct (x-axis). White bars are used for normal alleles, patterned for mutated constructs. Mutations within the same exons are grouped together and with their corresponding normal exon. Error bars represent standard deviation. The mutated constructs causing significant differences when data were analysed using Student's *t *test are underlined (* = P < 0.05, ** = P < 0.01,*** = P < 0.001).

**Figure 3:**
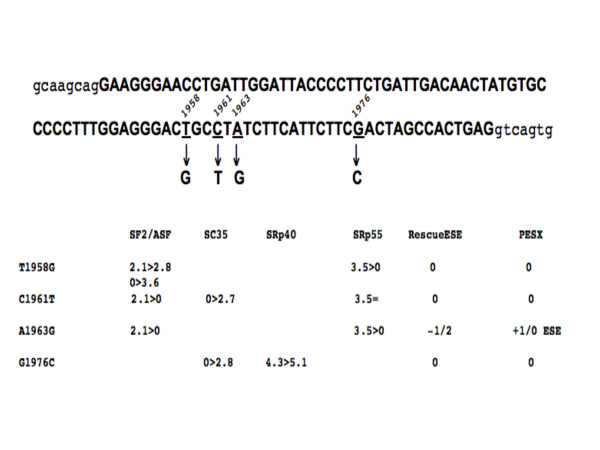
**Effects of the hMLH 1 exon 17 mutations on ESE sequences identified by the different algorithms**. The complete sequence of exon 17 is shown (exonic sequence in capital bold). Numbering is relative to the nucleotide position in the ORF. The four exon 17 mutations are shown, wild type sequence underlined. The consequences of the four mutations on the predicted motif scores identified by ESEfinder, RescueESE and PESX are shown below.

Figure 2A,B shows the results of the RT-PCR experiments on Cos-7 cells transfected with the 20 different mutations analyzed and their respective normal controls consisting of the corresponding non mutated exon. As a positive control for the splicing assay we used the C6354T mutation in exon 51 of the FBN1 gene, which has already been reported to cause exon skipping both in vivo and in vitro [20]. Overall, the results of this analysis showed that even if all the mutations fall in ESE sites predicted from either ESEfinder, or RescueESE or PESX, less than half of them led to splicing alterations. In particular, aberrant splicing should be expected whenever a mutation abrogates one or more ESE sites without creating novel sequence motifs recognized by other SR proteins. However, even when the mutations were clustered in a small exonic region, they demonstrated a splicing proficiency not always corresponding to the one expected on the basis of the algorithms predictions. Paradigmatic results were obtained from the splicing assay of the four different hMLH1 mutations T1958G, C1961T, A1963G and G1976C, that all lie in exon 17. The first two lead to concurrent creation of novel ESE sites, in addition to those already present in the wild type sequence, and disruption of one ESE motif for the SRp55 and one for the SF2/ASF splicing factor, respectively (see figure 3 and table 1). In fact, these two mutations did cause only slight changes to the ratio of exon inclusion compared to the normal exon (fig. 2). The A1963G mutation abrogated the same two ESE motifs, as well as one of the two ESEs predicted by RescueESE, but A1963G while not creating any novel ESE sequence for ESEfinder and RescueESE, did generate an ESE sequence according to PESX (fig. 3). The G1976C mutation added a novel ESE site to the one already identified by ESEfinder as present in the wild type sequence, while no ESE motifs are predicted in either the normal or the mutated allele by both RescueESE and PESX. Surprisingly, only the G1976 mutation dramatically altered the rate of exon inclusion in the splicing assay (figures 2, 3 and table 1), while the A1963G mutation, which should have been responsible for the most severe effect on splicing, according to both ESEfinder and RescueESE, instead caused an increase in the exon inclusion rate.

It has to be said that the splicing assay demonstrated that exon 17 inclusion was only partial for all of these mutations, as it was for the wild type exon. This situation mimics what has been observed in vivo for hMLH1, since alternative splicing of exon 17, where the four mutations are located, has been reported by several authors [21]. In addition, mutation G1976C and its cognate G1976A were already reported to lead to aberrant splicing in vivo [22]. Since this mutation does not alter any recognized enhancer or suppressor sequence, this data lend further support to the hypothesis of the presence of a different auxiliary element in this exonic region [23]. The other 5 mutations that in our splicing assay did change significantly the ratio of exon inclusion were C842T in hMLH1 exon 10, C806T and C815T in hMSH2 exon 5, and the two mutations in hMSH2 exon 10, G1516T and C1600T (fig. 2). The splicing behaviour was correctly predicted by PESX and ESEfinder for four of these five, while Rescue ESE correctly predicted the consequences on splicing of the only mutation localizing in a sequence identified as a splicing enhancer by this algorithm. Significantly all of these five mutations, as those in hMLH1 exon 17, lie in exons that have been reported to be alternatively spliced in vitro [21,24].

In conclusion, summarizing the results of the in vivo splicing analysis, 8 of the 20 mutations investigated caused a significant change in the splicing pattern and 6 led to a decrease of at least 50% of the rate of exon inclusion when compared to the wild type allele. The splicing consequences of these 8 mutations, correlated with ESEfinder predictions in 4 cases, 6 mutations were localized in ESE or ESS sequences identified by PESX which correctly predicted the splicing behaviour of 5 of them, while only 3 lie in ESE sequences recognized by RescueESE whose prediction correlated with the splicing behaviour of 2 of these 3. Furthermore, when exons were included or skipped completely in our assay they appeared to be insensitive to any change affecting ESE or ESS sequences. The higher correlation level of PESX predictions with the splicing pattern probably relies on the fact that, PESX differently from ESE finder and Rescue-ESE predicts not only exonic enhancer sequences but also those with a suppressor effect. Overall, the results of our splicing assay were comparable to the in vivo splicing profile of hMLH1 and hMSH2, since exons normally presenting alternative splicing all showed an inclusion rate ranging from null to 73%, but never complete.

### Analysis of the 20 mutations with NNSPLICE, SpliceSite finder and GENSCAN

The results of the transient transfection experiments demonstrated that the splicing pattern for the 20 selected mutations cannot be precisely predicted on the basis of ESEfinder, RescueESE and PESX computations. We then decided to investigate their effects using additional in silico resources, namely the two splice site prediction programs NNSPLICE [25] and SpliceSite finder [26], and the gene prediction program GENSCAN [27]. This latter program has been reported to reliably predict the splicing consequences of 4 different nucleotide substitutions in *MLH1 *and *BRCA1 *genes that caused an in vivo RNA splicing defect [28]. The results obtained with these further computer analyses are reported in table 1. Of the 99 different missense mutations listed in the InSIGHT mutation database, only 6 led to a relatively large change (mean change in probability p = 0.230, standard deviation ± 0.102, see additional file 1) of GENSCAN score. Of these, 2 were in terminal exons, 2 were in the splicing junctions (therefore having clear consequences on splicing), while 2 were located in internal exons, far from the splicing sites. These latter mutations, G731A and G1976C, were both analyzed in our assay. The hMLH1 G731A mutation, that causes a consistent change of GENSCAN score (see table 1), is in the hMLH1 exon 9 that is not recognized as an internal exon by GENSCAN and is skipped in the splicing assay regardless of the presence of the mutation. Finally mutation G1976C which caused the largest decrease of GENSCAN scores did lead to a significant increase in exon skipping (fig. 2A,B). On the contrary, none of the mutations analyzed introduced any significant change in the splicing donor (SD) and acceptor (SA) site scores identified by NNsplice or in those calculated by SpliceSite finder for the SD and SA+BPS sites.

### Alternative splicing and some mutations show a cell line specific effect

To investigate the possibility that some mutations may show tissue-specific differences, we used the same constructs analyzed in Cos-7 cells to transfect two other cell lines, namely the cervical adenocarcinoma-derived HeLa cell line and the hepatocellular carcinoma cell line Hep-3B. The RT-PCR results on RNA extracted from these two cell lines 48 hours after transfection with the different mutated and normal constructs demonstrated a variable level of inclusion for the exons already reported to be alternatively spliced in vivo (exons 2,3,5,6 and 10 for hMSH2, exons 10 and 17 for hMLH1).

**Figure 4:**
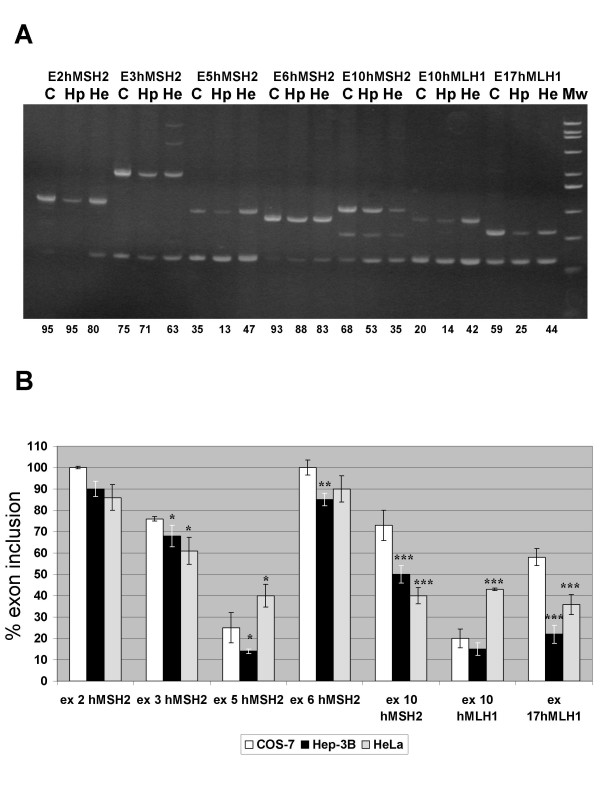
**Splicing assay mimics in vivo alternative splicing and varies in human cell lines**. (A) Results of the splicing assay following transfection with constructs harboring the different hMSH2 and hMLH1 normal exons. Cos-7 (C), Hep-3B (Hp) and HeLa (He) cells were transfected with 1 μg of the indicated wild-type exon, RNA was extracted, reverse transcribed, and amplified with primers SD6 and SA2. The RT-PCR products were resolved on GeneGel Excel, stained with ethidium bromide and quantitated with an image analyzer (see methods). Mw = size standard. The percentage of exon inclusion is indicated below each lane. (B) The graph shows the average of percent exon inclusion in the three different cell lines when transfected with the contructs containing the different normal exons (y-axis), representing the mean of two independent transfections done in triplicate for each construct (x-axis). Error bars represent standard deviation. The data were analysed using Student's *t *test (* = P < 0.05, ** = P < 0.01, *** = P < 0.001).

In particular, in the HeLa and Hep-3B cells, the hMSH2 exon 10 inclusion level was decreased by 30% and 45%, respectively, compared to the level observed in Cos-7 cells (fig. 4A,B). The hMSH2 exon 5 and the hMLH1 exon 10 both showed a large increase in the rate of exon inclusion in HeLa cells compared to Cos-7 (60 and 115%, respectively). Finally, the hMLH1 exon 17 inclusion was nearly halved in HeLa cells and decreased to one third in Hep-3B.

We next analyzed the consequences on splicing of the mutations that had been able to cause splicing abnormalities when tested in Cos-7 cells. Although for the majority of the mutations analyzed the effects on the splicing proficiency were similar to those observed in Cos-7 cells, for the mutations situated in the alternatively spliced exons there was an evident variability in the level of inclusion.

**Figure 5:**
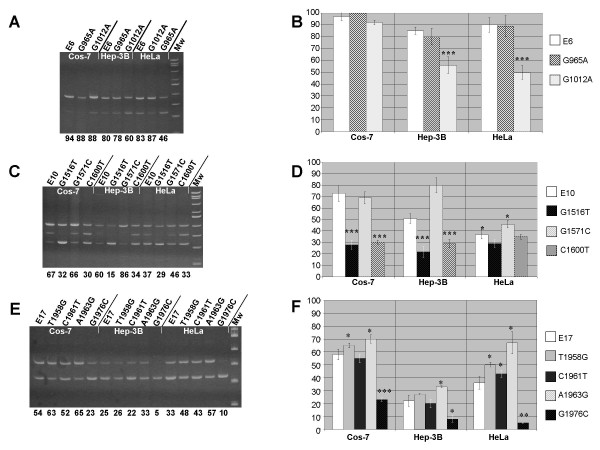
**Splicing effects of mutations in hMSH2 exons 6 and 10, and hMLH1 exon 17 in the three different cell lines**. Panels A (hMSH2 exon 6), C (hMSH2 exon 10), E (hMLH1 exon 17) show the RT-PCR results of the splicing assay performed in the three cell lines transfected with 1 μg of the mutated and corresponding normal constructs. 48 hours after transfection, RNA was extracted, reverse transcribed and amplified with primers SD6 and SA2. The RT-PCR products were resolved on GeneGel Excel, stained with ethidium bromide and quantitated with an image analyzer (see methods). Mw = size standard. The percentage of exon inclusion is indicated below each lane. The graphs (B for hMSH2 exon 6 and its mutations, D for hMSH2 exon 10 and its mutations, F for hMLH1 exon 17 and its mutations) show the averages of percent exon inclusion (y-axis) in the three different cell lines when transfected with the constructs containing the different mutated or normal exons, representing the mean of two independent transfections done in triplicate for each construct (x-axis). Error bars represent standard deviation. The data were analysed using Student's *t *test (* = P < 0.05, ** = P < 0.01, *** = P < 0.001).

The most dramatic change was observed for the mutations G965A and G1012A in exon 6 of the hMSH2 gene. When transfected in Cos-7 cells, these two mutations did not cause a change in the rate of exon inclusion compared to the normal allele, but in both Hep-3B and HeLa cells, G1012A led to a 40% decrease in exon inclusion (fig. 5A,B). Since this mutation disrupts all three ESE motifs identified by ESEfinder, it is possible that local changes in SR protein levels in both these two cell types cause the G1012A mutation to have an overt effect on splicing in Hep-3B and HeLa cells but not in Cos-7. Likewise, the hMSH2 exon 10 mutations G1516T and C1600T showed a decrease in the exon inclusion level by 70% in Cos-7 and by 28% in Hep-3B cells, but a very weak effect in the HeLa cellular background. The G1571C mutation in the same exon had no effect in Cos-7 cells, while leading to a 60% exon inclusion increase in Hep-3B and to a slight exon inclusion increase in HeLa (fig. 5C,D). A further example of cell-specific effects was observed for the four hMLH1 mutations in exon 17. All four mutations in this exon had a more severe effect when transfected in HeLa cells, compared to the consequences on splicing observed in Cos-7 and Hep-3B (fig. 5E,F). In fact, the G1976C mutation consistently reduced the ratio of exon inclusion in all the three cell lines but led to almost complete exon skipping exclusively in HeLa cells. C1961T caused a slight increase in exon inclusion only in HeLa cells, while T1958G and A1963G both increased exon 17 inclusion levels in all three cell lines, with a higher rate of inclusion in HeLa cells.

## Discussion

### Evaluation of putative splicing mutations by computer programs

Recent reports have shown that mutations in the coding region disrupting sequences recognized by splicing regulators such ESE, ESS or the recently identified composite exonic regulatory elements (CERES) [29], can be considered an additional mutation mechanism leading to disease in humans. This finding is particularly important for genetic counselling in HNPCC, where the pathogenicity assessment of any nucleotide substitution is crucial to correctly predict cancer risk.

Several experimental or computational approaches, aiming to identify regulatory sequence motifs whose mutations are predicted to alter splicing, have been developed. All of these experimental approaches share the functional evaluation of short random oligomer sequences in reporter systems represented by short exons with weak splicing sites. However, these functional assays have been performed in highly purified in vitro systems that may not fully reflect in vivo splicing conditions. In addition, these sequences with a putative enhancer activity have not been tested in their natural context, which can be represented by clusters of several overlapping motifs with complex and often antagonistic interactions as already demonstrated for the CFTR gene [29]. As a consequence, the ESE sequences predicted by the algorithms developed using these strategies tend to overlap with true splicing enhancers only when they lie in short, weakly defined exons. However, human exons are on average 130 bp in size and 99% of them possess strong, well-defined splicing sites. The preferential colocalization of pathogenic mutations with ESE sequences as compared with neutral polymorphisms has been reported [16] but a systematic evaluation of missense mutations and neutral polymorphisms predicted to alter splicing have not been performed.

The splicing analysis we have performed suggests that the ESEs predicted by these algorithms are likely to act as real enhancer when mutations fall in short loosely defined exons that are more frequently expected to contain sequences promoting exon inclusion in the mature transcript. In fact, most of the nucleotide changes associated with altered splicing, lie in exons averaging 67 bp in size, well below the 130 bp dimension of a typical human exon [10]. Furthermore, our data are supported from recent work [30] that shows that the ability to function as an enhancer is dependent on its natural surrounding environment and ESE position in the exon.

Hence, thorough in silico analysis and the knowledge of the alternative splicing profile of the gene of interest may contribute to assign pathogenic significance. In this study we have analyzed all the pathogenic missense mutations reported in the HNPCC mutation database, with three ESE prediction programs (ESEfinder, Rescue ESE and PESX), then with two splice site prediction programs (NNSPLICE and SpliceSite finder) and a gene prediction program (GENSCAN). Among the 99 mutations examined, 72 changed ESE motifs scores predicted by ESEfinder, RescueESE or PESX. Of these, 20 were analyzed for their splicing proficiency with an in vivo splicing assay we had already used in the past. Not surprisingly, the majority of mutations tested (12 of 20) did not alter the normal constitutive or alternative splicing pattern. Indeed, most exons in the hMSH2 and hMLH1 genes are large and well defined with few notable exceptions. It is worthy of note that all of the 8 mutations that did affect splicing in the assay we used fall in exons for whom alternative splicing has been reported, or showing suboptimal splice site scores when analyzed with NNSPLICE and SpliceSite finder (hMSH2 exons 5 and 10, hMLH1 exon 17), or in exons not recognized by GENSCAN (hMLH1 exon 10). Our data and a recent functional analysis performed on hMLH1 missense mutations [31] suggest that most pathogenic substitutions in MMR genes impair the biological function of the protein rather than splicing proficiency. Furthermore, when tested on mutations far from the splicing site, the prediction power of ESEfinder revealed a sensitivity lower than that recently reported [32].

### Differences in the alternative splicing profile and in the mutations effects between cell types

A large amount of data is accumulating, supporting the hypothesis that the global alternative splicing profile reflects tissue identity, and that alternative splicing acts independently on different sets of genes, defining tissue-specific expression profiles. The expression levels of antagonistic splicing factors, such as hnRNPA1 and SF2/ASF, have been shown to affect splice site selection [33], and colon cancer progression [34]. Thus, if a tissue-specific regulation of pre-mRNA splicing exists and depends on local differences of the regulatory factors concentration, it is likely that some mutations may show tissue- or cell-specific effects. However, the presence of such variability has not been thoroughly investigated. We therefore decided to investigate whether the mutations analyzed in our in vivo assay showed a different splicing profile in three different cell lines. An invariant pattern of splicing was observed for the constitutive exons but intriguingly for the mutations lying in alternative exons, both the mutated and the normal alleles showed variability in the ratio of exon inclusion. In fact, differences were observed both in the level of inclusion of alternative normal exons and in the magnitude of changes caused by mutations localizing in these exons (figs. 4, 5). These results confirm the recent findings based on microarray analysis, demonstrating that human tissues show rather divergent patterns of alternative splicing, yet correlated with differences in the splicing factor expression across tissues [35]. As a consequence, the net results of a mutation altering the splicing pattern might derive from local changes in the concentration of splicing modulators.

Both *MLH1 *and *MSH2 *genes show extensive alternative splicing with as many as eight different isoforms reported for *MLH1 *and seven for *MSH2 *[21,24]. More than half of these alternative isoforms are predicted to give rise to truncated proteins. Some of these isoforms have been reported to be expressed also in normal individuals and tissues [21] but no studies have assessed quantitatively the expression of these splice variants at the RNA level.

Our systematic analysis of different mutations in the hMLH1 and hMSH2 genes in three human cell lines, shows that a single mutation can provoke quite diverse consequences in different cell types. Similar results were obtained when the three cell lines were transfected with exons reported to be alternatively spliced in vivo, suggesting that cell specificity may nudge splice selection toward a variable ratio of exon inclusion vs skipping. In rapidly replicating tissues immunohistochemical analysis have demonstrated that MLH1/MSH2 proteins are highly expressed [36,37]. Therefore, in tissues where the requirements for protection from DNA replication errors are inherently higher, the relative abundance of isoforms with different MMR proficiency may be critical in determining the time and the frequency of cancer pathogenesis.

## Conclusion

Our study indicates that missense mutations in MLH1 and MSH2 are likely to affect splicing only when located in weak alternative exons. However when they do so, they might modulate in a tissue specific manner cancer onset and its phenotypic manifestations.

## Methods

### Generation of datasets and computational analysis

The 99 missense mutations analyzed were annotated in the InSIGHT database accessed in March 2003. All the exons of the *MLH1 *and *MSH2 *genes with the adjacent intronic sequences included in the construct used for the splicing assay (see below) were analyzed using the five software packages ESEfinder, RescueESE, NNSPLICE, SpliceSiteFinder, GENSCAN, and PESX. Only for GENSCAN analysis, was the whole genomic contig sequence of the *MLH1 *(NCBI accession number NT_022517.17) and *MSH2 *(NCBI accession number NT_022184.14) genes used to generate computer predictions. In all cases the score values were calculated for both normal and wild type allele.

### Mutagenesis and plasmid construction

Wild-type sequences of each exon used in the assay, as well as 50–160 nucleotides each side of flanking intronic sequences, always including the putative branch point site (identified with SpliceSite Finder), were amplified from human genomic DNA, using XhoI and BamHI tagged primers (primer sequences available on request). The 20 single nucleotide substitutions were introduced by overlap extension PCR with primers tagged with XhoI and BamHI restriction sites. All the 20 missense mutations analyzed and the respective controls consisting of the corresponding wild type exons in which the mutations were located, were cloned in the pSPL3 vector (fig. 1). All plasmid constructs were confirmed by DNA sequencing and in all cases the nucleotide substitution introduced represented the only difference between the mutated construct and its corresponding normal control.

### Transient transfection and analysis of RNA splicing pattern

All the transfections were performed in 24 well plates at approximately 90% cell confluence using Lipofectamine 2000 (Invitrogen). All the cell lines were grown in DMEM supplemented with 10% fetal bovine serum and 5 mM glutamine. Cells were subjected to a maximum of two passages in culture before transfection. For each transfection 1 μg of total plasmid DNA were used. cDNA synthesis and PCR were performed as previously reported [12], with the exception that 25 cycles were used to maintain the PCR in the logarithmic phase. For gel analysis the PCR reactions were resolved on precast polyacrylamide gels (GENEGEL EXCEL AP Biotech) stained with ethidium bromide. Gel images were obtained and the results quantitated using a Bio-Rad Versadoc 4000 imager system and Quantity One^© ^software. The identity of all the RT-PCR products was confirmed by sequencing.

## Authors' contributions

PL carried out all the mutagenesis, transfection and RT experiments and participated in the design of the study. NCS participated in the transfection and RT experiments. NR participated in the design of the study and the mutagenesis experiments. GG participated in the design of the study and helped to draft the manuscript. AS conceived and coordinated the study, participated in its design and drafted the manuscript. All authors read and approved the final manuscript.

## Note added in proof

Recently an upgrade of the alternative splicing database (ASD) has been published [38]. It is a collection of literature-based data set containing useful tools for splicing related analysis such as sequences and properties of alternatively spliced exons, characterization of observed splicing regulatory elements, a collection of minigene constructs and many more features.

## Supplementary Material

Additional File 1 Analysis of the hMSH2 (table A) and hMLH1 (table B) missense mutations. These two tables report the results of the analysis with the three softwares ESEfinder, RescueESE and PESX, of all the missense mutations listed in the InSIGHT mutation database. Click here for file

## Figures and Tables

**Table 1:** Pathogenic Missense Mutations analyzed in the splicing assay

					NNSPLICE^e^		SpliceSite Finder^f^					
									
Mutation^a^	Exon	ESEfinder^b^	Rescue ESE^c^	PESX^d^	SA	SD	SA+BPS	SD	GENSCAN Exon P-value^g^	Dist. from the nearest SS	Exon size (bp)	**Mutation effect on splicing**
**hMSH2**												
A308G	2	SRp40 3.05>0	0	0	0.99	1	170.8	85.6	0.998(0.998)	-58	155	**=**
A593G	3	SF2 3.67>2.80 +SRp40	-2/2	0	0.98	1	*161.5*	94.2	0.861(0.861)	-52	279	**=**
**C806T**	**5**	**SRp55 2.67>0**	**-1/1**	**+1/0 PESS**	**0.96**	**0.92**	**NI**	**79.9**	**0.987(0.985)**	**+14**	**150**	↓
**C815T**	**5**	**SF2 3.2>0 SRp55 2.7>0**	**0**	**-1/1 PESS**	**0.96**	**0.92**	**NI**	**79.9**	**0.987(0.985)**	**+23**	**150**	↓
G965A (3)	6	SC35 3.39>3.03 SRp40 5.72>3.34	0	+1/0 PESE	0.98	0.98	187.8	88.7	0.999(0.999)	+24	134	**=**
G1012A	6	SF2 2.2>0 SC35 2.6>0 SRp404.3>0	+2/5	-1/1 PESE	0.98	0.98	187.8	88.7	0.999(0.999)	-64	134	**=**
**G1516T**	**10**	**SC35 5.44>4.79 + SC35**	**0**	**-4/4 PESE**	**NI**	**0.60**	**150.5**	**72.8**	**0.991(0.991)**	**+6**	**151**	↓
G1571C	10	SRp55 3.3>0 + SC35	0	0	NI	0.60	150.5	72.8	0.991(0.991)	+61	151	**=**
**C1600T**	**10**	**SRp55 3.4>0**	**0**	**+1/0 PESS**	**NI**	**0.60**	**150.5**	**72.8**	**0.991(0.991)**	**-61**	**151**	↓
**hMLH1**												
G199A (8)	2	SF2 4.26>2.34 +1SF2 +1 SC35	0	0	0.86	1	180.4	92.2	0.790/0.789	-8	91	**=**
G200A (2)	2	SF2 4.26>0	-1/7	0	0.86	1	180.4	92.2	0.790/0.789	-7	91	**=**
T320G	4	SRp55 3.3 >0 + SF2 2.8	+4/1	+3/0 PESE	1	0.88	155.6	69.7	0.987/0.990	+14	74	**=**
G731A	9	SRp40 3.7>0	0	-3/3 PESS	0.88	0.96	156.4	*78.3*	*0.338/0.205 term*	+54	113	**=**
C793T (2)	10	SRp40 3.85>4.2	0	0	0.99	0.93	167.8/171	81.0	NI/NI	+4	94	**=**
**C842T**	**10**	**SF2 3.2>0 SC35 2.6>0 SRp55 4.7>3.1+SRP55**	**0**	**= 1 PESS**	**0.99**	**0.93**	**167.8**	**81.0**	**NI/NI**	**-42**	**94**	↓
G1569T	14	0	-3/3	0	0.98/0.97	0.99	182.3	85.6	0.912/0.976	+11	109	**=**
**T1958G**	**17**	**SF2 2.1= SRp55 3.5>0 + SF2**	**0**	**0**	**NI**	**0.91**	**166.6**	**90.0**	**0.836/0.877**	**-31**	**93**	↑
C1961T	17	SF2 2.1>0 SRp55 = +SC35	0	0	NI	0.91	166.6	90.0	0.836/0.828	-28	93	**=**
**A1963G**	**17**	**SF2 2.1> 0 SRp55 3.5>0**	**-1/2**	**+1/0 PESE**	**NI**	**0.91**	**166.6**	**90.0**	**0.836/0.857**	**-26**	**93**	↑
**G1976C (3)**	**17**	**SRp40 4.3>5.15+SC35**	**0**	**0**	**NI**	**0.91**	**166.6**	**90.0**	**0.836/0.490**	**-13**	**93**	↓

^a ^in parenthesis the number of families/cases in the database presenting the mutation.

^b ^effect of the mutation on the scores as predicted by the algorithm.

^c ^number of ESE motifs added or abrogated from the mutation/number of ESE motifs in the normal allele.

^d ^number of enhancer (PESE) or suppressor (PESS) sequences added or abrogated from the mutation/number of PESE or PESS in the normal allele.

^e ^SA = splice acceptor, SD = splice donor, NI = not identified as corresponding splicing site by the program, in italics are indicated the suboptimal scores.

^f ^SA+BPS = splice acceptor + branch point sites, NI = not identified as corresponding splicing site by the program, in italics are indicated the suboptimal scores.

^g ^in parenthesis the score for the wild type allele; term = the exon is recognized as a terminal exon.

In bold the mutations significantly altering splicing: = no change in exon inclusion; ↑ = increase in exon inclusion; ↓ = increase in exon skipping.
