# Supplementary material for: In silico and in vivo splicing analysis of MLH1 and MSH2 missense mutations shows exon- and tissue-specific effects
Source: BMC Genomics. 2006 Sep 22;7:243. doi: 10.1186/1471-2164-7-243 (PMC1590028; doi:10.1186/1471-2164-7-243)
Supplement: Additional File 1 — Analysis of the hMSH2 (table A) and hMLH1 (table B) missense mutations. These two tables report the results of the analysis with the three softwares ESEfinder, RescueESE and PESX, of all the missense mutations listed in the InSIGHT mutation database. [file 1471-2164-7-243-S1.doc]

**SUPPLEMENTARY TABLE A**

Analysis of the InSIGHT pathogenic missense mutations for the *hMSH2* Gene

| **Mutation^a^** | **ESE Finder^b^** | **Rescue^c^**  **ESE** | **PESX^d^** | **Exon** | **Dist from the closest SS^e^** | **Exon size (bp)** | **AA consequence** | **Type of AA Sub^f^** |
| --- | --- | --- | --- | --- | --- | --- | --- | --- |
| G4A | 0 | 0 | 0 | 1 | -207 | 211 | A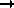T | C |
| A182C | SRp40 2.87>0 | 0 | -1/1 PESE | 1 | -29 | 211 | Q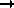P | C |
| **A308G** | **SRp40 3.05>0** | **0** | **0** | **2** | **-58** | **155** | **Y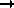C** | **R** |
| G319C | 0 | 1/1= | =1PESE | 2 | -47 | 155 | A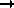P | C |
| A380G (5) | 0 | 0 | +1/0 PESE | 3 | +14 | 279 | N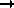S | C |
| T435G (2) | SRp40 3.33>0 | 0 | 0 | 3 | +69 | 279 | I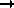M | C |
| G446A | 0 | +1/0 | +1 PESS | 3 | +79 | 279 | G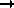D | R |
| A505G | SC35 3.35>3.80 | 0 | 0 | 3 | -140 | 279 | I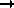V | C |
| **A593G** | **SF2 3.67>2.80 , + SRp40** | **-2/2** | **0** | **3** | **-52** | **«** | **E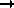G** | **R** |
| T595C | 0 | -2/2 | -1/1 PESS | 3 | -50 | « | C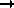R | R |
| A742G | 0 | +1/2 | 0 | 4 | -50 | 147 | L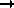E | C |
| G792C | Splicing site | -1/2 | 0 | 4 | 0 | 147 | Q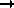H | R |
| **C806T** | **SRp55 2.67>0** | **-1/1** | **+1/0 PESS** | **5** | **+14** | **150** | **S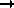L** | **R** |
| **C815T** | **SF2 3.2>0, SRp55 2.7>0** | **0** | **-1/1 PESE** | **5** | **+23** | **150** | **A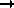V** | **C** |
| G913A | SC35 3.00>2.55, SRp55 4.71>4.10  +SRp40 | 0 | 0 | 5 | -28 | 150 | A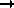T | C |
| **G965A (3)** | **SC35 3.39>3.03, SRp40 5.72>3.34** | **0** | **+1/0 PESE** | **6** | **+24** | **134** | **G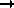A** | **C** |
| T997C | 0 | -1/1 | 0 | 6 | +56 | 134 | C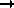R | R |
| G998A | 0 | 1/1= | 0 | 6 | +57 | 134 | C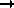Y | R |
| **G1012A** | **SF2 2.2>0, SC35 2.6>0,**  **SRp40 4.3>0** | **+2/5** | **-1/1 PESE** | **6** | **-64** | **134** | **G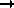R** | **R** |
| A1082G | 0 | 0 | 0 | 7 | +6 | 200 | R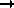S | R |
| T1319C | SRp40 3.38>3.02 | 0 | 0 | 8 | +43 | 110 | L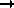P | R |
| T1508C | 0 | 0 | =7 PESE | 9 | -2 | 124 | L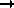P | R |
| **G1516T** | **SC35 5.44>4.79**  **+ SC35** | **0** | **-4/4 PESE** | **10** | **+6** | **151** | **D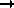Y** | **R** |
| **G1571C** | **SRp55 3.3>0, + SC35** | **0** | **0** | **10** | **+61** | **«** | **R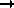P** | **R** |
| **C1600T** | **SRp55 3.4>0** | **0** | **+1/0 PESS** | **10** | **-61** | **«** | **R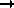C** | **R** |
| A1774G | SC35 2.58>2.95, SRp40 3.31>3.15  +SRp55 | 2/2= | +1/0 PESE | 12 | +15 | 246 | M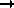V | C |
| G1807A | 0 | -1/1 | 0 | 12 | +48 | « | D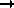N | R |
| C1864A | 0 | +1/0 | -2/3 PESE | 12 | +105 | « | P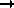T | R |
| C1865T | 0 | +1/0 | -3//3 PESE | 12 | +106 | « | P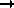L | R |
| G1906A (4) | SRp40=, SRp55 3.6>0 | 2/2= | 0 | 12 | -99 | « | A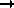P | C |
| G2090T (2) | 0 | 0 | 0 | 13 | +85 | 205 | C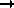F | R |
| G2164A | SF2 2.09>4.02 | 3/3= | 0 | 13 | -46 | « | V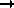I | R |
| G2251A | 0 | +2/1 | +3/4 PESE | 14 | +41 | 248 | G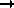R | R |
| G2500A | 0 | 0 | 0 | 15 | +42 | 176 | A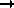T | C |
| C2714 G | SRp40 3.3>0, SRp55= | 0 | 0 | 16 | +80 | 171 | T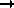R | R |
| C2714 T | SRp40 3.3>0, SRp55= | 0 | 0 | 16 | +80 | « | T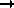R | R |
| A2790G | 0 | +3/0 | -1/1 PESE | 16 | +156 | « | I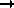M | C |

 NOTE.
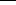
The table lists missense mutations from the InSIGHT mutation database (accessed in March 2003). **a** the numbering is with reference to the ORF, in parenthesis the number of different patients/families reported with the mutation; **b**  0= the mutation is not localized in an ESE motif, = no score change introduced by the mutation, + = creation of a novel ESE motif; **c**  number of ESE motifs added or abrogated from the mutation/ number of ESE motifs in the normal allele, 0= the mutation is not localized in an ESE motif; **d** number of enhancer (PESE) or suppressor (PESS) sequences added or abrogated from the mutation/number of PESE or PESS in the normal allele;  **e** the number indicates distance from splice donor (negative) or from splice acceptor (positive); **f** C = conservative; R = radical. In bold the mutations selected for the splicing analysis.

**SUPPLEMENTARY TABLE B**

Analysis of the InSIGHT pathogenic missense mutations for the *hMLH1* Gene

| **Mutation^a^** | **ESE Finder^b^** | **Rescue^c^**  **ESE** | **PESX^d^** | **Exon** | **Dist from the closest SS^e^** | **Exon size (bp)** | **AA consequence** | **Type of AA Sub^f^** |
| --- | --- | --- | --- | --- | --- | --- | --- | --- |
| A69T | 0 | -1/1 | +1/0 PESS | 1 | -47 | 116 | E 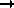D | C |
| A73T | 0 | 0 | 0 | 1 | -43 | « | I 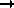 F | R |
| T74C | 0 | 0 | 0 | 1 | -42 | « | I 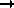T | R |
| C83T | SC35=, +SRp55 | 0 | 0 | 1 | -33 | « | P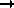 L | R |
| G85T | 0 | 0 | 0 | 1 | -31 | « | A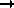S | C |
| T104G | 0 | -2/4 | +3/1 ESE | 1 | -12 | « | M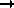R | C |
| G116A | Splicing junction | -1/3 | -1/1 PESE | 1 | 0 | « | C 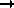Y | R |
| C184A | SC35 3.22>2.46 | +3/8 | 0 | 2 | -23 | 91 | Q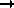L | C |
| A191G | SRp40 3.46>3.30 | -2/8 | 0 | 2 | -16 | « | N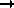S | C |
| **G199A (8)** | **SF2 4.26>2.34**  **+1SF2 +1SC35** | **0** | **0** | **2** | **-8** | **«** | **G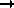R** | **C** |
| **G200A (2)** | **SF2 4.26>0** | **-1/7** | **0** | **2** | **-7** | **«** | **G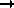E** | **R** |
| T203A | 0 | -2/7 | +1/0 PESS | 2 | -4 | « | I 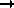N | R |
| G230A | 0 | +2/1 | -1/0 PESS | 3 | +23 | 99 | C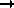Y | R |
| A250G | 0 | +2/0 | 0 | 3 | +43 | « | L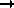E | R |
| A277G | 0 | 0 | 0 | 3 | -29 | « | S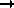G | R |
| G299C | SC35 3>0 | 0 | 0 | 3 | -7 | « | R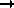P | C |
| G304A | SRp55 >0 | 0 | 0 | 3 | -2 | « | E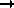L | R |
| G306T | SRp55 >0 | 0 | 0 | 3 | 0 | « | E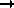D | R |
| **T320G** | **SRp55 3.3 >0, + SF2 2.8** | **+4/1** | **+3/0 PESE** | **4** | **+14** | **74** | **I 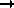R** | **R** |
| C350 T (11) | SRp40 4.6>3 | -2/4 | -1/1 PESE | 4 | -30 | « | T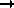M | R |
| G382C | SF2 3.2=, SRp40 2.7>0 | 0 | -1/1 PESE | 5 | +1 | 73 | A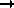P | C |
| C479T | (SF2, SRp40)=, +SRp40 | 0 | 0 | 6 | +26 | 92 | A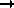V | R |
| T554G | 0 | +2/1 | 0 | 7 | +11 | 43 | V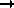G | R |
| T577C | 0 | 0 | 0 | 7 | +32 | « | S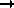P | C |
| G595C | 0 | -1/3 | -1/3 PESE | 8 | +50 | 89 | E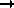Q | R |
| G637 A(4) | 0 | 0 | +1/0 PESE | 8 | -40 | « | V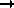M | C |
| C649 T(2) | 0 | 0 | 0 | 8 | -28 | « | R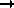C | R |
| G677 A(3) | Splicing junction | +3/2 | 0 | 8 | 0 | « | R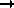Q | R |
| **G731A** | **SRp40 3.7>0** | **0** | **-3/3 PESS** | **9** | **+54** | **113** | **G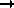D** | **R** |
| A791G | 0 | 0 | 0 | 10 | +2 | 94 | R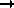D | R |
| **C793T (2)** | **SRp40 3.85>4.20** | **0** | **0** | **10** | **+4** | **«** | **R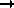C** | **R** |
| G794A | SRp40 3.8>0, SRp55 3.2= | 0 | +1/0 PESE | 10 | +5 | « | R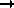H | C |
| A803G | 0 | 4/4= | 0 | 10 | +14 | « | E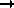G | R |
| T814G | 0 | 0 | 0 | 10 | +25 | « | L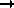V | R |
| **C842T** | **SF2 3.2>0, SC35 2.6>0, SRp55 4.7>3.1**  **+SRp55** | **0** | **=1 PESS** | **10** | **-42** | **«** | **A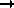V** | **R** |
| T977C (3) | SRp55 2.44>, +SF2, +SRp55 | 0 | 0 | 11 | -61 | 154 | V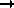A | R |
| A986C | SRp55 4>0, +SF2 2.8 | 0 | 0 | 11 | -52 | « | H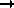P | C |
| G1166A | (SC35, SRp40)=  +SF2+SC35+SRp40 | +2/0 | +1/1 PESE | 12 | +128 | 371 | R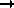Q | C |
| G1217A | 0 | +2/0 | +1/0 PESE | 12 | +179 | « | S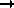N | C |
| G1321A (2) | SF2=,SC35=,+SRp40 | 0 | 0 | 12 | -88 | « | A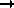T | R |
| G1421A | 0 | +4/2 | +1/4 PESE | 13 | +12 | 149 | R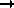Q | R |
| G1474A | (SF2,SC35,SRp40)=  +SRp40 | +1/1 | 0 | 13 | +65 | « | A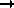T | R |
| T1517C (3) | 0 | 0 | -1/1 PESS | 13 | -41 | « | V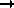A | R |
| **G1569T** | **0** | **-3/3** | **0** | **14** | **+11** | **109** | **E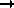D** | **R** |
| A1625T | SF2 3.4>0, SRp40 =  SRp55 3.3>0, SRP55= | 0 | -1/1 PESE | 14 | -42 | « | Q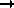L | R |
| T1646C | 0 | 0 | 0 | 14 | -21 | « | L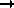P | R |
| A1652T | SRp40 3.9>0, +SRp40 | +1/0 | 0 | 14 | -15 | « | D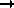T | R |
| A1693T | 0 | 0 | 0 | 15 | +26 | 64 | I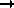F | R |
| T1721C | (SF2, SC35, SRp40) =, +SC35 | 0 | -1/1 PESE | 15 | -10 | « | L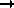P | R |
| A1733G | 0 | 0 | -1/1 PESE | 16 | +2 | 165 | E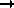G | R |
| C1744G | 0 | 0 | 0 | 16 | +13 | « | L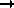V | C |
| *A1771G* | *0* | *Pos*  *Err?.* |  | *16* | *40* | « | *E*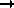*G* | *R* |
| A1853C | 0 | -2/3 | -1/7 PESE | 16 | -43 | « | K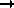T | R |
| **T1958G** | **SF2=, SRp55 3.5>0, + SF2** | **0** | **0** | **17** | **-31** | **93** | **L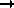R** | **R** |
| **C1961T** | **SF2 2.1>0, SRp55 =, +SC35** | **0** | **0** | **17** | **-28** | **«** | **P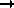L** | **R** |
| **A1963G** | **SF2 2.1> 0, SRp55 3.5>0** | **-1/2** | **+1/0 PESE** | **17** | **-26** | **«** | **I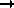V** | **C** |
| **G1976C (3)** | **SRp40 4.33>5.15 , +SC35** | **0** | **0** | **17** | **-13** | **«** | **R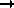P** | **C** |
| A1988G | SF2 4.26>2.80, SRp40 3.34>5.71  +SC35 | 0 | -1/1 PESE | 17 | -1 | « | E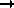G | R |
| T2027G | 0 | 0 | 0 | 18 | +38 | 114 | L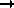R | R |
| G2041A (2) | 0 | “ | 0 | 18 | +51 | 114 | A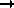T | R |
| G2146A (2) | SF2 2.16>0 , SC35= | “ | -2/2 PESE  +1/0 PESS | 19 | +43 | 168 | V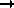M | C |
| C2152T | 0 | 2/2= | 0 | 19 | +49 | 168 | H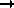Y | R |

 NOTE.The table lists missense mutations from the InSIGHT mutation database (accessed in March 2003). **a** the numbering is with reference to the ORF, in parenthesis the number of different patients/families reported with the mutation; **b**  0= the mutation is not localized in an ESE motif, = no score change introduced by the mutation, + = creation of a novel ESE motif; **c**  number of ESE motifs added or abrogated from the mutation/ number of ESE motifs in the normal allele, 0= the mutation is not localized in an ESE motif; **d** number of enhancer (PESE) or suppressor (PESS) sequences added or abrogated from the mutation/number of PESE or PESS in the normal allele; **e** the number indicates distance from splice donor (negative) or from splice acceptor (positive); **f** C = conservative; R = radical. In bold the mutations selected for the splicing analysis.
